# Evaluation of Cartosat-1 Multi-Scale Digital Surface Modelling Over France

**DOI:** 10.3390/s90503269

**Published:** 2009-04-29

**Authors:** Marco Gianinetto

**Affiliations:** Politecnico di Milano, Remote Sensing Laboratory, BEST Department, Piazza Leonardo Da Vinci 32, 20133 Milano, Italy; E-Mail: marco.gianinetto@polimi.it; Tel. +39-02-23996204; Fax: +39-02-23996550

**Keywords:** Satellite remote sensing, Cartosat-1, digital surface model generation, accuracy assessment, C-SAP

## Abstract

On 5 May 2005, the Indian Space Research Organization launched Cartosat-1, the eleventh satellite of its constellation, dedicated to the stereo viewing of the Earth's surface for terrain modeling and large-scale mapping, from the Satish Dhawan Space Centre (India). In early 2006, the Indian Space Research Organization started the Cartosat-1 Scientific Assessment Programme, jointly established with the International Society for Photogrammetry and Remote Sensing. Within this framework, this study evaluated the capabilities of digital surface modeling from Cartosat-1 stereo data for the French test sites of Mausanne les Alpilles and Salon de Provence. The investigation pointed out that for hilly territories it is possible to produce high-resolution digital surface models with a root mean square error less than 7.1 m and a linear error at 90% confidence level less than 9.5 m. The accuracy of the generated digital surface models also fulfilled the requirements of the French Reference 3D®, so Cartosat-1 data may be used to produce or update such kinds of products.

## 1. Introduction

On 5 May 2005, the Indian Space Research Organization (ISRO) launched Cartosat-1, the eleventh satellite of its IRS constellation, dedicated to the stereo viewing of the Earth's surface [1,2]. Cartosat-1 carries two high-resolution imaging cameras: the afterward looking camera (Aft) and the foreword looking camera (Fore), both able to collect panchromatic images with a spatial resolution of 2.5 m on the ground. The imaging cameras are fixed to the spacecraft to acquire near-simultaneous imaging of the same scene (with a delay of 52 s between the Fore and the Aft acquisitions) from two different angles: +26° with respect to nadir for the Fore camera and -5° with respect to nadir for the Aft camera. This configuration is optimized for along-the-track stereo data collection in a 30 km swath and with a base-to-height ratio of 0.62. However, Cartosat-1 is also able to collect 2.5 m mono images with a combined swath of 55 km [3]. The satellite was mainly designed for terrain modeling and large-scale mapping [3,10-16]. Nevertheless, in previous studies Cartosat-1 data have been also used in different fields, such as natural hazards assessment [4,5], archaeological exploration [6], estimation of hydrological parameters [7,8] or estimation of atmospheric aerosols [9].

In early 2006, ISRO started the Cartosat-1 Scientific Assessment Programme (C-SAP) jointly established with the International Society for Photogrammetry and Remote Sensing (ISPRS). The aim of the C-SAP was to assess the mapping capabilities of the Cartosat-1 satellite for different types of terrain and for different applications, such as photogrammetric stereo triangulation at scene and block level, extraction of terrain features, terrain modeling and topographic mapping. For this purpose, ISRO and ISPRS selected thirty research groups from different countries as C-SAP Investigators (seventeen from Europe, seven from Asia, five from USA and Canada and one from South America) and provided them with Cartosat-1 stereoscopic data collected over eleven test sites (seven in Europe, one in Asia, one in USA and one in Australia), along with metadata, ground control points (GCPs) and reference digital surface models (DSMs).

Within the C-SAP framework, Politecnico di Milano University (Italy), being one of the European Investigators, evaluated the capabilities of terrain modeling for the French test sites of Mausanne les Alpilles and Salon de Provence. The investigation focused on the generation of multi-resolution DSMs and was addressed to typical remote sensing users (also for non specialists in photogrammetry). Since the study would like to be able to provide some operative hints about the potentialities and limits in the generation of DSMs from Cartosat-1 data for landscapes similar to the C-SAP French test sites, all the data processing was done using standard commercial off-the-shelf software (RSI ENVI^®^) rather than homemade or scientific software.

Results were compared with reference data expressly acquired for C-SAP [15,17] and also with existing standards and products actually used in France (i.e., the French Institut Géographique National's and Spot Image's Reference 3D^®^, the French DB Alti^®^ and the French DB ORTHO^®^). Finally, the investigation also provided a comparison between the Cartosat-1 DSMs and the global Shuttle Radar Topography Mission (SRTM) DSMs, widely used in the remote sensing community as topographic layer [18].

## 2. Results and Discussion

Generally speaking, we can affirm that the Cartosat-1 DSM's accuracy decreases as the number of GCPs used decreases, with increasing ground sampling distance and with increasing terrain slope. Moreover, the use of high quality GCPs is fundamental to obtain good DSMs, filtering may help to enhance the elevation accuracy and the generation method used is fundamental for determining the final quality of products. Following, the effect of each of them will be considered.

### 2.1. Influence of GCPs

The influence of GCPs in the generation of absolute DSMs was studied by analyzing dozens of 25 m resolution DSMs generated for both Mausanne les Alpilles and Salon de Provence using different GCPs number and configuration. Their accuracy was validated both locally using Independent Check Points (ICPs) and on the whole study areas using the reference DSMs/DTMs supplied by the Principal Investigators. With respect to ICPs, for Mausanne les Alpilles the best results were achieved using five GCPs (four in the corners and one in the centre), obtaining a mean value of residuals (μ) of -0.0 m, a standard deviation (σ) of 1.7 m and a RMSE of 1.7 m. For Salon de Provence, the best results were achieved using nine GCPs regularly distributed obtained a mean value of residuals of 0.5 m, a standard deviation of 1.4 m and a RMSE of 1.2 m. We should note, however, that similar results were achieved using fewer GCPs: using four GCPs we obtained a RMSE of 1.3 m, while using six GCPs we found a RMSE of 1.2 m. Consequently, for the test field studies we can affirm that the sensor orientation can be carried on with at least least GCPs. This outcome has been also confirmed by other studies handling the same dataset or other datasets [15-17,43,48-50].

NATO standards [38] suggest the use for evaluation control of data more than twice as accurate as the expected final DSMs. Nevertheless, the accuracy assessment based on a reduced number of spot elevations (ICPs) is biased and not realistic and tends to overestimate the real accuracy [27]. For that reason, we also compared the Cartosat-1 DSMs with reference DSMs/DTMs on the whole test fields to obtain more reliable results. For Mausanne les Alpilles, passing from only one GCP to five GCPs the improvement in the computation of the DSM's accuracy was +34% in terms of RMSE. No further significant enhancement was observed using more than nine GCPs (data referred to the high-resolution reference DSM). For Salon de Provence, passing from only one GCP to four GCPs the improvement in the computation of the DSM was +12% in terms of RMSE, while passing from four GCPs to nine GCPs the improvement was only +6% in terms of RMSE. Again, no further significant enhancement was observed using more GCPs (data referred to MNT DBTOPO^®^ DTM).

The reason for the accuracy difference obtained using only one against four (or more) GCPs is due to the use of RPCs for image orientation. Data processed without any GCPs mainly show linear systematic errors and few GCPs can be used to improve the positioning accuracy by fitting the RFM calculated coordinates to the coordinates of the additional GCPs; with one GCP is possible only to correct for shifts while using more GCPs an additional transformation in the image space can be applied (e.g., 2-D affine transformation) [51].

### 2.2. Influence of ground sampling distance

The influence of the DSM's ground sampling distance is one of the factors that influence the quality of the generated models. A recent study involving SPOT-HRV and Terra ASTER data [30] described the best resolution as twice the satellite's image pixel size to avoid degradation of the DSM's structure (i.e., 20 m for SPOT-HRV and 30 m for Terra ASTER).

When evaluating the multi-resolution DSMs generated at the desired resolution directly from the Cartosat-1 images in the range of 5 – 20 m, for Mausanne les Alpilles we observed a RMSE between 7.1 m and 9.4 m, while for Salon de Provence we obtained a RMSE between 4.5 m and 5.1 m. When analyzing results in the range of 25 – 85 m resolution, for Mausanne les Alpilles we observed a RMSE between 7.9 m and 12.4 m, while for Salon de Provence we obtained a RMSE between 5.0 m and 11.7 m with respect to the high-resolution reference DSM and a RMSE between 7.1 m and 14.7 m with respect to MNT DBTOPO® DTM. When analyzing results at the SRTM90 resolution (90 m), for Mausanne les Alpilles we obtained a RMSE of 7.1 m, while for Salon de Provence a RMSEs of 10.3 m and 15.3 m, respectively comparing to the high-resolution reference DSM and MNT DBTOPO^®^ DTM.

**Figure 1.**
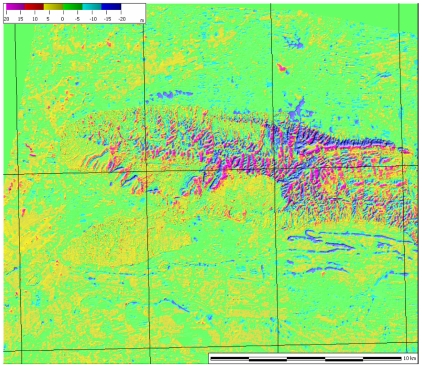
Residuals for the 10 m resolution DSM generated over Mausanne les Alpilles (France).

**Figure 2.**
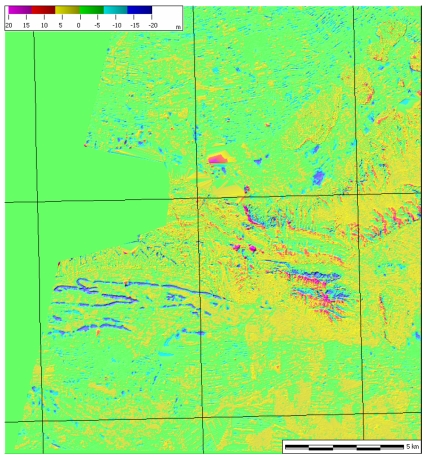
Residuals for the 10 m resolution DSM generated over Salon de Provence (France).

**Figure 3.**
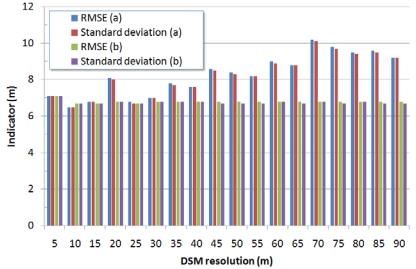
Summary of results for the Cartosat-1 multi-resolution DSMs generated over Mausanne les Alpilles test site. Comparison with respect to the high-resolution reference DSM (reference DSM-Cartosat-1 DSM): (a) for DSMs generated at the desired resolution directly from the Cartosat-1 images; (b) for DSMs generated by downsampling the 5m resolution Cartosat-1 DSM. All values refer to a comparison over the whole test site.

**Figure 4.**
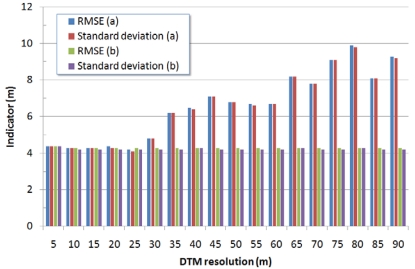
Summary of results for the Cartosat-1 multi-resolution DSMs generated over Salon de Provence test site. Comparison with respect to the high-resolution reference DSM (reference DSM-Cartosat-1 DSM): (a) for DSMs generated at the desired resolution directly from the Cartosat-1 images; (b) for DSMs generated by downsampling the 5m resolution Cartosat-1 DSM. All values refer to a comparison over the whole test site.

When evaluating the multi-resolution DSMs generated by downsampling higher-resolution models previously generated (5 m) we obtained a remarkable improvement for both the test sites (Figures 1 and 2) and the DSMs' accuracy did not depend neither upon the ground sampling distance nor upon the terrain's slope and was almost constant. The overall accuracy for all the multi-resolution DSMs showed a RMSE between 6.8 m and 7.1 m for Mausanne les Alpilles and a RMSE between 4.3 m and 4.4 m for Salon de Provence (Figures 3 and 4). A more surprising improvement was achieved when comparing the multi-resolution Cartosat-1 DSMs with Reference 3D^®^ specifications. In this case Reference 3D^®^ requirements were always met, regardless the DSM's resolution and the terrain's slope (Tables 1 and 2). As a drawback, this method is disadvantageous in terms of computation time and resources since a higher-resolution DSM must be computed to produce a lower-resolution terrain model.

### 2.3. Influence of terrain's slope

With regards to the dependence of the DSM's vertical accuracy and the terrain's slope, for both the test sites we observed an increase of residuals as the terrain's slope increased. Referring to LE90, for Mausanne les Alpilles it was between 4.9 m and 8.7 m for slopes lower than 20%, between 13.7 m and 25.8 m for slopes included in 20% and 40% and between 20.0 m and 30.7 m slopes greater than 40%. For Salon de Provence our investigation assessed a value between 4.7 m and 8.4 m for slopes lower than 20%, between 6.8 m and 25.5 m for slopes included in 20% and 40% and between 9.2 m and 32.3 m slopes greater than 40%. Tests clearly show higher slope corresponding to lower elevation accuracy. The reason for this may be due to the presence of more errors on slopes and, above all, to the use of different interpolation methods for the generation of Cartosat-1 DSM grids and reference DSMs/DTMs grids from the original matched or measured points.

### 2.4. Influence of data smoothing

Digital surface models derived from imagery are typically represented using triangular irregular networks (TINs) or regular grids. When producing gridded DSMs, the matched points (scattered) are interpolated on a regular grid and often have a noisy pattern caused by the combined effect of various types of errors and natural surface roughness and need to be further processed [52]. Data smoothing is a technique widely used to remove peaks and smooth down (regularize) the generated surfaces [53,54].

The use of a moving average low-pass filter with 7 × 7 kernel size in post-processing slightly improved the results. When evaluating the multi-resolution Cartosat-1 DSMs in the range of 5 – 20 m, for Mausanne les Alpilles we observed an improvement in the RMSE between 0% for 5 m resolution and +16.0% for 20 m resolution. For Salon de Provence we obtained an improvement in the RMSE between +3.9% for 10 m resolution and +15.9% for 20 m resolution. When analyzing results in the range of 25-85 m resolution, for Mausanne les Alpilles we observed an improvement in the RMSE between +0.5% for 85 m resolution and +26.7% for 45 m resolution. For Salon de Provence we recorded an improvement of the RMSE between -11.1% (a decrease) for 85 m resolution and +19.0% for 25 m resolution with respect to the high-resolution reference DSM, and an improvement between +4.9% for 65 m resolution and +20.5% for 85 m resolution with respect to MNT DBTOPO^®^ DTM. Finally, when analyzing results at the SRTM90 resolution, for Mausanne les Alpilles we obtained a worsening of results (-23.1% for RMSE), while for Salon de Provence we obtained again an improvement of results (+10.8% for RMSE when compared to the high-resolution reference DSM and +19.5% for RSME when compared to MNT DBTOPO^®^ DTM).

### 2.5. Comparison of results with other Cartosat-1 investigations on the same test sites

The outcomes of this study are in large parts comparable with those obtained by other investigators using the same dataset. Regarding the DSM's generation for the test field of Salon de Provence, Gachet and Favé [17] described for 30 m resolution Cartosat-1 DSMs a RMSE between 6.5 m and 9.5 m when comparing results to MNT DBTOPO^®^ DTM. Krishna Murthy *et al.* [39] assessed for 10 m resolution Cartosat-1 DSMs a mean value of residuals of -3.3 m and a standard deviation of 7.5 m. Similarly, the same authors [39] reported for 10 m resolution Cartosat-1 DSMs a mean value of residuals of -0.4 m and a standard deviation of 7.0 m when checking results with the SRTM DSM. Lehner *et al.* [49], using only excellent points from hierarchical matching in the generation process, reported a mean of height differences to the reference DSM of -1.5 m and standard deviation of 2.2 m for 10-meters Cartosat-1 DSMs. The same authors [49] assessed a mean of height differences to the reference DSM of -1.1 m and standard deviation 3.5 m when using all the points from obtained with region growing.

Regarding the DSM's generation for the test field of Mausanne les Alpilles, Titarov [48] produced 10-meters grid cell size Cartosat-1 DSMs with the following statistics: RMSE = 7.2 m, LE90 = 14.1 m, mean error = 0.8 m and mean absolute error = 4.4 m. Similarly to Salon de Provence, Lehner *et al.* [49], using only excellent points from hierarchical matching in the generation process, reported a mean of height differences to the reference DSM of -1.9 m and standard deviation of 2.2 m for 10-meters DSMs. Moreover, the same authors [49] assessed a mean of height differences to the reference DSM of -1.4 m and standard deviation 3.8 m when using all the points from obtained with region growing. Baltsavias *et al.* [46] in their investigation concluded that Cartosat-1 data have a good potential for the generation of DSMs with a grid spacing of about 10 m and reported a RMSE of 3 m. However, it is important to note that the authors found this result investigating only a reduced area of the Mausanne les Alpilles test site and not the whole test site, so results may be better than those of other investigators.

Finally, for both Salon de Provence and Mausanne les Alpilles test sites, Lehner *et al.* [49] also described an improved technique which, using a multi-ray (4-ray) forward intersection for the overlapping area of the test fields, was able to give Cartosat-1 DSMs a mean value of residuals of -1.3 m and a standard deviation of 3.4 m.

### 2.6. Results from other Cartosat-1 studies

Considering different test sites, terrain types and elevation ranges, Cartosat-1 proved to be a very good source of data for DSM modeling. Michalis and Dowman [16] reported for the test field of Aix-en-Provence (France) a RMSE less than 7 m for 15-meters Cartosat-1 DSMs. Nandakumar *et al.* [40] found height differences in DSMs generated from MTF enhanced Cartosat-1 images up to 10 m for both Hobart (Australia) and Castel Gandolfo (Italy) test sites, respectively at 91.7% and 93.1% confidence level. Lehner *et al.* [41] investigated the generation of digital terrain models for different test sites using region growing for densification: they found a standard deviation for elevation residuals of 3.1 m with a bias in elevation of -1.0 m for the Catalonia (Spain) test field and a standard deviation for elevation residuals of 7.3 m, with a -3.6 m bias in elevation for a reduced area of the Bavaria (Germany) test fields. Srivastava *et al.* [47] reported an elevation accuracy for Cartosat-1 DSMs generated with point spacing of 10 m and evaluated for different types of terrain all over India better than 5 m LE90. Titarov [48] investigating the Warsaw (Poland) test site showed that the Cartosat-1 derived DSMs are extremely close to the reference DSMs/DTMs: for 20-meters DSMs he found a RMSE of 2.3 m (estimated LE90 of 4.5 m), a mean error of 1.0 m and a mean absolute error of 1.7 m. For the same test site (Warsaw), Michalis and Dowman [50] reported a LE90 between 3.88 m and 4.07 m.

Finally, regarding the generation of high-resolution Cartosat-1 DSMs with 5 m spacing for the flat area of the Roma (Italy) test site (elevation range of only 40 m), Crespi *et al.* [42] reported a RMSE of about 2 – 3 m, while Sadasiva Rao *et al.* [43] discussed, for the same test site (Roma), a LE90 of 3.4 m. These last two investigations, however, considered only the urban area of Roma and results were verified comparing the heights of some buildings and spots on road axes with respect to 1:2,000 3D map, while all other differences between the generated DSMs and reference data were not taken into account. So results are not directly comparable with the outcomes of other investigations.

### 2.7. Comparison between Cartosat-1 DSMs and DSMs generated using other high resolution satellites

More in general, Cartosat-1 stereo images have proven to be an excellent source of data for the production of DSMs with a ground resolution of about 10 m. Even if within the range of available high resolution optical remote sensing satellites there are several units with a higher geometric resolution than Cartosat-1, Cartosat-1 DSMs can nevertheless be compared to similar models produced from higher resolution input imagery. For example, using IKONOS data Poon *et al.* [44] reported for the Hobart (Australia) test field a RMSE of about 4 m for urban areas and a RMSE of about 5 m for forests when comparing results with high-resolution first-pulse LIDAR data (elevation accuracy of reference data of 0.25 m). Zhang and Gruen [45] found a RMSE of about 5 m for DSMs generated using IKONOS data collected over the test field of Thun (Switzerland), again using as reference data an accurate laser DSM (elevation accuracy of reference data of 0.5 m).

Cuartero *et al.* [31] analyzed the generation of 10 m resolution DSMs using SPOT-HRV images collected over the test site of Granada (Spain) and found a RMSE between 7.7 m and 8.6 m when validating results using GCPs.

Finally, Toutin [22] compared 10-meters DSMs generated using SPOT-5, EROS-A, IKONOS and QuickBird data with very high-resolution LIDAR data (elevation accuracy of reference data of 0.15 m) acquired over the test site of Québec City (Canada). He found a LE90 of 9 m for QuickBird DSMs, a LE90 of 10 m for IKONOS DSMs and SPOT-5 DSMs and a LE90 of 31 m for EROS-A DSMs.

## 3. Experimental Section

### 3.1. Theoretical background

The theoretical relative accuracy of DSMs produced from satellite's images can be computed, as a first approximation, using a simplified one-dimensional model which takes into account the measured x-parallax and the B/H ratio [37]:

(1) $$\Delta h(x,y)\approx \left(\frac{\Delta p(x,y)}{B/H}\right)+{\left(\frac{\Delta p(x,y)}{B/H}\right)}^{2}\frac{1}{H}$$

where:

Δh(x,y) is the relative height for the generic image point (x,y);
Δp(x,y) is the parallax for the generic image point (x,y);
B/H is the base-to-height ratio;
H is the height of the sensor above the DSM.

For high flying altitudes, such as those of remote sensing satellites, the second term of the right side of equation 1 can be neglected, thus the relative height can be computed as:

(2) $$\Delta h(x,y)\approx \left(\frac{\Delta p(x,y)}{B/H}\right)$$

Since x-parallax measurements can be obtained with automatic matching with an error of about ±0.5 – 1 pixels [27], given the Cartosat-1 B/H ratio of 0.62 and its pixel spacing of 2.5m, for null y-parallax residual of the quasi-epipolar images Δh(x,y) is in the range of 2 – 4 m. Moreover, in addition we have a limited influence of the sensor orientation, approximately between 1 and 5 m for Cartosat-1 data depending upon the terrain type, the number, configuration and accuracy of GCPs used [15-17,43,48-50]. For real applications we should expect larger errors both due to the y-parallax residual and possible x-parallax measurements with lower accuracy.

### 3.2. Study areas and dataset

**Figure 5.**
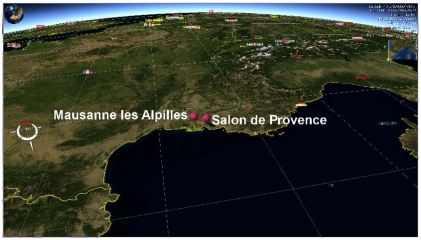
French C-SAP test fields of Mausanne les Alpilles and Salon de Provence (view made with NASA World Wind^®^).

The investigation was carried on in the heart of Provence (France), in the triangle of Arles, Avignon and Marseille (Figure 5), in the Mausanne les Alpilles and Salon de Provence C-SAP test sites. For Mausanne les Alpilles, an area of about 650 km^2^ was selected as study area (centre image coordinates: 4,844,944 m North and 644,291 m East, projection: UTM, datum: WGS84). Here ground elevation ranges from 47m in the south-west to 633m in the north-east and the territory presents a mixture of rural areas (60%), green forests (35%) and urban areas (5%). For Salon de Provence, an area of about 600 km^2^ was selected as study area (centre image coordinates: 4,847,651 m North and 666,697 m East, projection: UTM, datum: WGS84). Like Mausanne les Alpilles, the test field is hilly, with elevations ranging from 83 m in the south-west to 710 m in the north-east and presents a mixture of rural and urban areas. Figure 5 shows an overview of the two study areas.

**Figure 6.**
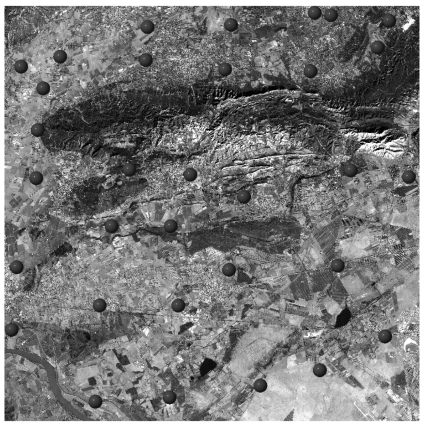
Cartosat-1 Aft image collected over Mausanne les Alpilles (France) with superimposed the high-precision GPS measured GCPs provided by the Principal-Investigator European Commission-Joint Research Centre.

**Figure 7.**
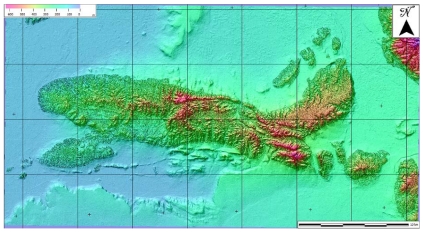
High-resolution raster DSM for Mausanne les Alpilles (France), purpose-made with a Leica ADS40 digital camera and provided by the Principal-Investigator European Commission-Joint Research Centre.

**Figure 8.**
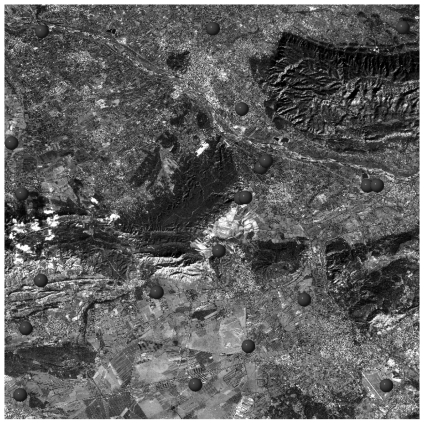
Cartosat-1 Aft image collected over Salon de Provence (France) with superimposed the GCPs derived from the French DB ORTHO® database provided by the Principal-Investigator French Institut Géographique National.

The standard C-SAP dataset of Mausanne les Alpilles delivered to the study teams was composed of a 2.5 m Cartosat-1 stereo pair collected by ISRO on 31 January 2006, a set of 32 high-precision GPS measured GCPs (Figure 6) and a 2 m ground resolution DSM purpose-made with a Leica ADS40 digital camera (Figure 7). Both GCPs and the DSM were provided by the Principal-Investigator European Commission-Joint Research Centre. Similarly, the standard C-SAP dataset of Salon de Provence was composed of a 2.5 m Cartosat-1 stereo couple collected by ISRO on 6 February 2006, a set of 22 GCPs derived from the French DB ORTHO® database (Figure 8) and a medium-resolution digital terrain model (DTM) with 25 m ground resolution and part of the French MNT DBTOPO® DTM. Both GCPs and the DTM were provided by the Principal-Investigator French Institut Géographique National.

The Cartosat-1 stereoscopic data were delivered by ISRO as GeoTIFF Stereo Orthokit product with a location accuracy of 220 m (three-sigma tolerance) [20,21]. ISRO also supplied to the study teams predetermined Rational Polynomial Coefficient (RPCs), based on its own orbit and attitude model for the acquisition, and metadata for the correction of the imagery geometric distortions [22-25].

Regarding the vertical accuracy of reference data, the European Commission-Joint Research Centre supplied for Masuanne les Alpilles GCPs with a vertical accuracy of 0.05 m and a high-resolution DSM with a root mean square error (RMSE) of 0.6 m [15]. The French Institut Géographique National supplied for Salon de Provence GCPs with a vertical accuracy better than 2.5 m (geolocation error less than 1.5 m) and a medium-resolution DTM with a 1 m RMSE, evaluated over the whole France [17].

In addition to this dataset, we also used for comparison the radar DSM generated from the NASA's SRTM mission and available free of charge from the NASA JPL's ftp server [19]. In terms of linear error at 90% confidence level (LE90), the SRTM DSM generated for Eurasia is accredited of an absolute elevation accuracy of 6.2 m and an absolute geolocation error of 8.8 m [26].

### 3.3. Methods

Digital terrain models are one of the most important sources of data used in geomatics and geoscientific analysis. The topographic information is required for many of the geometric, radiometric and atmospheric corrections of satellite data, both for optical and microwave instruments, and the production of orthoimages, more and more used in applications, also requires elevation data in the form of DSMs/DTMs [27]. Low and medium-resolution global DSMs are easily available free of charge, such as the GTOPO30 DSM [28] or the SRTM DSM (SRTM30 for USA and SRTM90 worldwide) [29], but high-resolution DSMs with suitable detail are still not available for much of the Earth's surface, and, when available, they do not always have enough accuracy for engineering and environmental applications [27].

This study investigated terrain modeling using the high-resolution images captured by Cartosat-1 and commercial off-the-shelf software (RSI ENVI^®^), the influence of the GCPs in the generation process [30], the influence of the sampling interval and of the terrain's slope on the quality of the generated models [31,32] and the influence of data processing on final results. Here we do not used procedures to filter vegetation and man-made infrastructures, so DSMs were generated. Moreover, the Cartosat-1 DSMs have not been edited for blunders (manually or automatically) as other authors reported in their investigations [50]. Even if in the practical production a check and editing for blunder should be performed, our goal was to test the performances of the DSMs generation using full automatic data processing (with the only exclusion of GCPs selection).

The software used allowed to quickly create both relative DSMs, as well as absolute DSMs, from stereo images using the spacecraft's metadata. When creating absolute DSMs the terrain's elevations are referred to a geodetic datum through some GCPs, while for relative DSMs the terrain's elevation are referred to an arbitrary reference height (e.g., the lowest value in the scene) and no GCPs are needed. The generation of DSMs was carried out by means of automatic image matching [25]. The input Aft and Fore images were first transformed into a pair of quasi-epipolar images using RPCs and tie points. Quasi-epipolar images differ from true epipolar images because the differences between y coordinates of two corresponding image points (y-parallax) is small but not null, as occurs in true epipolar images [33]. However, as true epipolar images they show a displacement in the satellite flight direction (x-parallax) proportional to the terrain elevation, thus elevation data can be computed. Corresponding ground features were extracted using a coarse-to-fine hierarchical solution (image pyramids) with an image matching algorithm based on the Förstner operator [34]: matching features were found on the Aft and Fore images of the stereo pair, using the quasi-epipolar geometry to reduce the search space [35], and their displacements were transformed into elevation values. Finally, raster DSMs were generated by interpolating the elevation values computed and they were re-projected in a map coordinate system using few GCPs (only for absolute DSMs).

One of the major factors determining the accuracy of the generated DSMs, as well as the processing time needed for their computation, is the number of image pyramids used during the image matching. When using few image pyramids the processing time is very short but the number of matched points may be very limited and the DSM's quality poor. When using many image pyramids the number and quality of matched points increase, thus the overall quality of the generated DSMs, but at the expenses of the computation time. Although the computational power of the modern computers is impressively increasing [36], the calculation of high-resolution DSMs for large territories using ordinary workstations is still limited by system resources, therefore, from an operational point of view, the data processing must be optimized. As metadata we used the predetermined RPCs supplied by ISRO along with sixty tie points automatically extracted with a regular grid scheme. Under these conditions, the y-parallax of the quasi-epipolar images was 1.27 pixel (corresponding to 3.18 m on the ground). Any improvement in the DSMs' accuracy was observed using more tie points.

The image matching was done using a moving window size of 30 pixels and a minimum correlation coefficient of 0.80 and data processing was stopped just before the highest pyramid level (at full resolution): this led to a reduction of the computing time by a factor of 4.6 (size of images four times smaller) without sensible degradation in the DSMs.

For geocoding, we investigated how the GCPs' number and geometric distribution influenced the product's accuracy, while the influence of GCPs' accuracy on the produced DSMs was not tested because the dataset supplied within C-SAP never allowed such validation.

Finally, we verified that the DSM's generation process may be enhanced by applying a simple moving average low-pass filter with optimized kernel size (7 × 7 pixels).

Dozens of DSMs were generated for resolution ranging from 5 m to 90 m and their vertical accuracy has been manually evaluated with respect to the terrain's characteristics and sampling interval. Regarding the generation process we used two different approaches: first we generated DSMs at the desired resolution directly from the Cartosat-1 images (here resampling was performed as a part of the data processing) and then we generated DSMs by downsampling the 5 m resolution Cartosat-1 DSMs previously generated. The main difference of the two methods concerns how filtering was applied. In the first case, filtering was applied to the downsampled data, thus involved a ground surface depending on the DSM's resolution. In the second case, filtering was always applied to the higher-resolution DSM (5 m), and then downsampling was performed.

Results were evaluated with respect to the following reference data:

- A set of ICPs extracted from the original C-SAP dataset (ranging from to 23 to 31 for Mausanne les Alpilles and from 13 to 21 for Salon de Provence). The vertical accuracy of reference ICPs was the same of source dataset (0.05 m for Mausanne les Alpilles and better than 2.5 m for Salon de Provence);
- The high-resolution reference DSM resampled to the Cartosat-1 DSM's resolution;
- The medium-resolution reference DTM resampled to the Cartosat-1 DSM's resolution (used for comparison at resolution exceeding 25 m);

Another term of comparison used for evaluating the quality of the generated DSMs were the requirements for the French Reference 3D^®^: a 1 arc second DSM (∼30m on the Equator, ∼ 21 m at 45° of latitude) produced from SPOT-5/HRS satellite's stereoscopic data by IGN and Spot Image. Its specifications are an absolute horizontal CE90 of 15 m and an elevation absolute LE90 of 10 m for slopes lower than 20%, 18 m for slopes included in 20% and 40% and 30 m for slopes greater than 40% [17]. Finally, the 90 m resolution DSMs were also compared to the low-resolution SRTM90 DSM available for Eurasia.

The comparison of the Catrosat-1 models and reference data was carried on as a 2.5D comparison, where the difference in heights was computed under the hypothesis of same geolocation for the Cartosat-1 generated and reference DSMs. The accuracy of Cartosat-1 DSMs was tested in terms of both first and second order statistics of residuals (mean and standard deviation), RMSE and LE90, all of them computed from data and not derived under hypothesis of Gaussian distribution of data.

Since no characterization of the test sites (e.g., landscape, land cover/land use, vegetation, buildings, *etc.*) was available to the investigating teams for the study, all the comparisons have been performed on the complete dataset, evaluating results on the full range of field range, elevation range and land cover. This approach can give a clear idea of results achievable in real conditions but will not reach the best results obtainable when processing specific land covers (e.g., open areas, forest areas or urbanized areas) or limited elevation ranges (e.g., flat areas or hilly areas with small elevation range).

## 4. Conclusions

Within the Cartosat-1 Scientific Assessment Programme the accuracy of multi-resolution DSMs generated for the French test sites of Mausanne les Alpilles and Salon de Provence was investigated. The investigation revealed that when the data processing is performed using standard commercial off-the-shelf software and when few GCPs are available with a vertical accuracy better than 2.5 m and geolocation error less than 1.5 m, for hilly territories (with an elevation range of about 600 m) it is possible to produce high-resolution DSMs (up to 5 m grid spacing) with a RMSE less than 7.1. These values confirm those obtained by other investigating teams within the C-SAP and are also comparable with those obtained by other authors using different high-resolution satellite data. The accuracy of the generated DSMs proved to fulfill the requirements of the French Reference 3D^®^, so Cartosat-1 data may be used to produce such kind of product.

For applications requiring low-resolution DSMs, Cartosat-1 DSMs performed better than the SRTM90 for Salon de Provence and similarly to SRTM90 for Mausanne les Alpilles. If data are available, Cartosat-1 DSMs can be successfully used to fill refine and fill gaps in SRTM DSMs. A more interesting application would be the refinement of SRTM 1 arc second DSMs (∼ 30 m resolution), which is only available for the USA.

The limits of the study are mainly related to the non homogeneous nature of the C-SAP dataset: only for the Mausanne les Alpilles test field were available high accuracy GCPs and reference DSM, while for Salon del Provence were delivered low accuracy GCPs and reference DTM. As a matter of fact, for Salon de Provence the absolute geolocation was performed with a lower accuracy and this may have affected the overall DSMs generation process. Moreover, no land cover information for the test sites was available to the investigation teams, so it was not possible to carry on an accuracy evaluation based on different land cover types.

Comparing results to those described in literature, different authors may use different statistics for the evaluation process (e.g., mean of residuals, standard deviation, RMSE, LE90 or other statistics) computed on ground control points, independent check points, reference DTMs/DSMs, cartography, reduced study areas or something else. Consequently, results obtained by different authors, even on the same test site and using the same input data, may not always be easy to compare. Nevertheless, also considering all these limits, this study confirmed the outcomes of the other independent investigating teams and also confirmed the good quality of Cartosat-1 DSMs with relation to those generated using other high-resolution satellite data.

## Figures and Tables

**Table 1.** Summary of residuals vs. slope for Cartosat-1 DSMs generated over Mausanne les Alpilles test site. (a) for DSMs generated at the desired resolution directly from the Cartosat-1 images. (b) for DSMs generated by downsampling the 5m resolution Cartosat-1 DSM. All values refer to a comparison over the whole test site.

**Resolution** **(m)**	**LE90 (m) vs. slope ^(a)^**			**LE90 (m) vs. slope ^(b)^**		
	**< 20%**	**20% – 40%**	**> 40%**	**< 20%**	**20% – 40%**	**> 40%**
5	5.6	14.3	20.1	5.6	14.3	20.1
10	5.2	13.7	22.4	5.6	13.8	23.3
15	5.8	14.3	20.3	5.6	14.4	20.4
20	5.6	20.6	26.2	5.7	15.5	21.1
25	4.9	15.3	20.0	5.6	14.3	19.9
30	5.2	17.7	22.3	5.8	15.6	21.1
35	5.3	19.3	24.2	5.6	14.5	20.1
40	5.6	19.6	23.9	5.7	15.4	20.9
45	6.0	21.4	26.4	5.6	14.2	20.0
50	6.4	21.1	25.8	5.7	15.5	21.0
55	5.9	20.5	25.1	5.5	13.9	20.1
60	7.2	23.3	27.8	5.8	15.5	21.4
65	6.6	22.5	26.3	5.5	14.1	20.2
70	8.7	25.8	30.7	5.7	15.5	21.2
75	7.8	24.4	28.7	5.6	14.2	20.2
80	8.0	25.5	28.7	5.7	15.5	20.9
85	7.6	24.3	28.1	5.5	14.5	19.8
90	7.6	24.6	28.1	5.8	15.8	21.1

**Table 2.** Summary of residuals vs. slope for Cartosat-1 DSMs generated over Salon de Provence test site. (a) for DSMs generated at the desired resolution directly from the Cartosat-1 images. (b) for DSMs generated by downsampling the 5m resolution Cartosat-1 DSM. All values refer to a comparison over the whole test site.

**Resolution** **(m)**	**LE90 (m) vs. slope ^(a)^**			**LE90 (m) vs. slope ^(b)^**		
	**< 20%**	**20% – 40%**	**>40%**	**< 20%**	**20% – 40%**	**> 40%**
5	5.3	6.8	9.2	5.3	6.8	9.2
10	5.0	8.2	11.5	5.2	7.4	10.4
15	5.8	7.0	9.8	5.2	7.3	10.1
20	4.7	9.8	12.4	5.2	7.4	10.3
25	5.0	14.0	23.0	5.1	7.4	10.1
30	4.8	12.1	14.7	5.2	7.4	10.6
35	5.1	15.1	20.1	5.2	7.3	10.1
40	5.5	16.5	21.2	5.2	7.5	11.2
45	5.6	17.3	23.0	5.2	7.3	10.0
50	5.4	17.6	22.6	5.2	7.4	10.4
55	5.3	16.7	21.3	5.2	7.3	10.0
60	5.5	18.2	21.3	5.2	7.6	10.8
65	6.5	20.6	26.4	5.2	7.4	9.9
70	6.2	20.1	25.7	5.2	7.4	10.3
75	7.0	22.7	30.0	5.2	7.3	9.8
80	8.4	25.5	32.3	5.2	7.3	10.8
85	5.8	20.4	25.2	5.2	7.3	9.8
90	8.1	24.6	31.0	5.2	7.7	10.7
